# Trends and predictors of HIV positivity and time since last test at voluntary counselling and testing encounters among adults in Kilifi, Kenya, 2006-2017

**DOI:** 10.12688/wellcomeopenres.15401.3

**Published:** 2021-06-04

**Authors:** Peter M. Mugo, Clara A. Agutu, Elizabeth Wahome, Margaret Juma, Joseph Nzioka, Khamisi Mohamed, Teresia Mumba, Mahmoud Shally, Ibrahim Fauz, Anisa Omar, Tobias F. Rinke de Wit, Elise M. van der Elst, Susan M. Graham, Eduard J. Sanders

**Affiliations:** 1 Kenya Medical Research Institute (KEMRI) -Wellcome Trust Research Programme, Kilifi, Kenya; 2 Ministry of Health, Kilifi, Kenya; 3 Amsterdam Institute for Global Health and Development (AIGHD), Department of Global Health, University of Amsterdam, Amsterdam, The Netherlands; 4 Departments of Global Health, Medicine, and Epidemiology, University of Washington, Seattle, WA, USA; 5 Nuffield Department of Medicine, University of Oxford, Headington, UK

**Keywords:** Voluntary counselling and testing, HIV retesting, HIV diagnosis, Kilifi County, Kenya

## Abstract

**Background:** Little is known about HIV retesting uptake among key populations (KP) and general populations (GP) in Kenya. We assessed trends and predictors of first-time testing (FTT), late retesting (previous test more than one year ago for GP or three months for KP), and test positivity at three voluntary counselling and testing (VCT) centres in coastal Kenya.

**Methods**: Routine VCT data covering 2006-2017 was collected from three VCT centres in Kilifi County. We analysed HIV testing history and test results from encounters among adults 18-39 years, categorized as GP men, GP women, men who have sex with men (MSM), and female sex workers (FSW).

**Results:** Based on 24,728 test encounters (32% FTT), we observed declines in HIV positivity (proportion of encounters where the result was positive) among GP men, GP women, first-time testers and MSM but not among FSW. The proportion of encounters for FTT and late retesting decreased for both GP and KP but remained much higher in KP than GP. HIV positivity was higher at FTT and late retesting encounters; at FSW and MSM encounters; and at encounters with clients reporting lower educational attainment and sexually transmitted infection (STI) symptoms. HIV positivity was lower in GP men, never married clients and those less than 35 years of age. FTT was associated with town, risk group, age 18-24 years, never-married status, low educational attainment, and STI symptoms. Late retesting was less common among encounters with GP individuals who were never married, had Muslim or no religious affiliation, had lower educational attainment, or reported STI symptoms.

**Conclusions:** HIV positive test results were most common at encounters with first-time testers and late re-testers. While the proportion of encounters at which late retesting was reported decreased steadily over the period reviewed, efforts are needed to increase retesting among the most at-risk populations.

## Introduction

Kenya has the fifth-largest human immunodeficiency virus (HIV) epidemic in the world ^1^, with 1.3 million adults living with HIV in 2018 ^2^. Data from sentinel surveillance and national population-based surveys indicate that national HIV prevalence peaked at 10%–11% in the mid-1990s and declined to about 6% in 2006 ^1, 3, 4^. Prevalence has remained relatively stable at that level for several years with a modest decline observed from 2010 to 2017 ^5^. In 2018, national prevalence was estimated at 4.9%, higher in women (6.6%) than men (4.5%) ^2^. The epidemic is geographically diverse, with prevalence ranging from 19.6% in Homa Bay county in the west to <0.1% in Garissa county in the north-east ^2^. There were approximately 36,000 new infections in 2018 ^2^, with more than a third occurring among young women 15–24 years ^5, 6^. Key populations, including men who have sex with men (MSM) and female sex workers (FSW) remain disproportionately affected by HIV. In 2017, prevalence was estimated at 18% among MSM and 29% among FSW ^6^. County-level prevalence estimates for key populations are not available.

The proportion of Kenyan adults 15–64 years who have ever tested for HIV increased from 37% in 2007 to 70% in 2012 ^4, 7^, and to 80% in 2014 ^8^. This tremendous increase in testing coverage is the result of an expanded testing program, including voluntary counselling and testing (VCT), routine (opt-out) provider-initiated testing in health facilities, routine testing in prevention of mother-to-child transmission programs, home-based (door-to-door) testing, mobile testing, and annual testing campaigns. However, knowledge of HIV status remains low. In 2018, it was estimated that 79.5% of people living with HIV knew their status ^2^. This falls short of the UNAIDS target of 90% and plays a major role in ongoing transmission ^9^. It is estimated that 54–90% of new transmission events arise from persons with undiagnosed infection ^10– 13^.

Low knowledge of HIV status may be attributable in large part to infrequent testing. Current national HIV testing guidelines recommend retesting quarterly for key populations (KP) and annually for the general population (GP) ^14^. In 2012, a population survey estimated national retesting uptake at 55% among all adults 15–64 years ^7^. A more up-to-date estimate is not available, but a repeat survey was underway in 2018. Little is known about retesting uptake at the sub-national level or the factors that predict adherence to recommended retesting frequency. To address such information gaps, data collected at VCT centres can supplement population-based surveys ^15, 16^, if regularly and rigorously analysed. Currently, test data collected at various testing facilities are reported to county and national headquarters only in summary form, combining VCT and other testing points, and not disaggregated by risk groups.

Kilifi county, one of the six counties in the coastal region of Kenya, is among the poorest counties in Kenya ^17^, with low literacy levels and high rates of school dropout affecting both girls and boys ^18, 19^. In 2017, 30,597 adults were living with HIV in the county, for an estimated HIV prevalence of 3.8% ^5^. In the same year, the county experienced 1,380 new infections, with a third occurring among adolescents and young people in the age-group 15–24 years ^5^.

In the present study, we used routine data collected over a period of 12 years at three VCT centres located in three neighbouring towns in Kilifi county, to assess trends in HIV positivity (proportion of test encounters where the result was positive), and the proportion of encounters at which clients reported first-time testing (never tested before) or late retesting (previous test more than one year ago for GP or three months for KP). Information on these outcomes in different sub-groups who utilize VCT services can support the targeting of HIV prevention efforts.

## Methods

### Study setting and population

**Figure 1.**
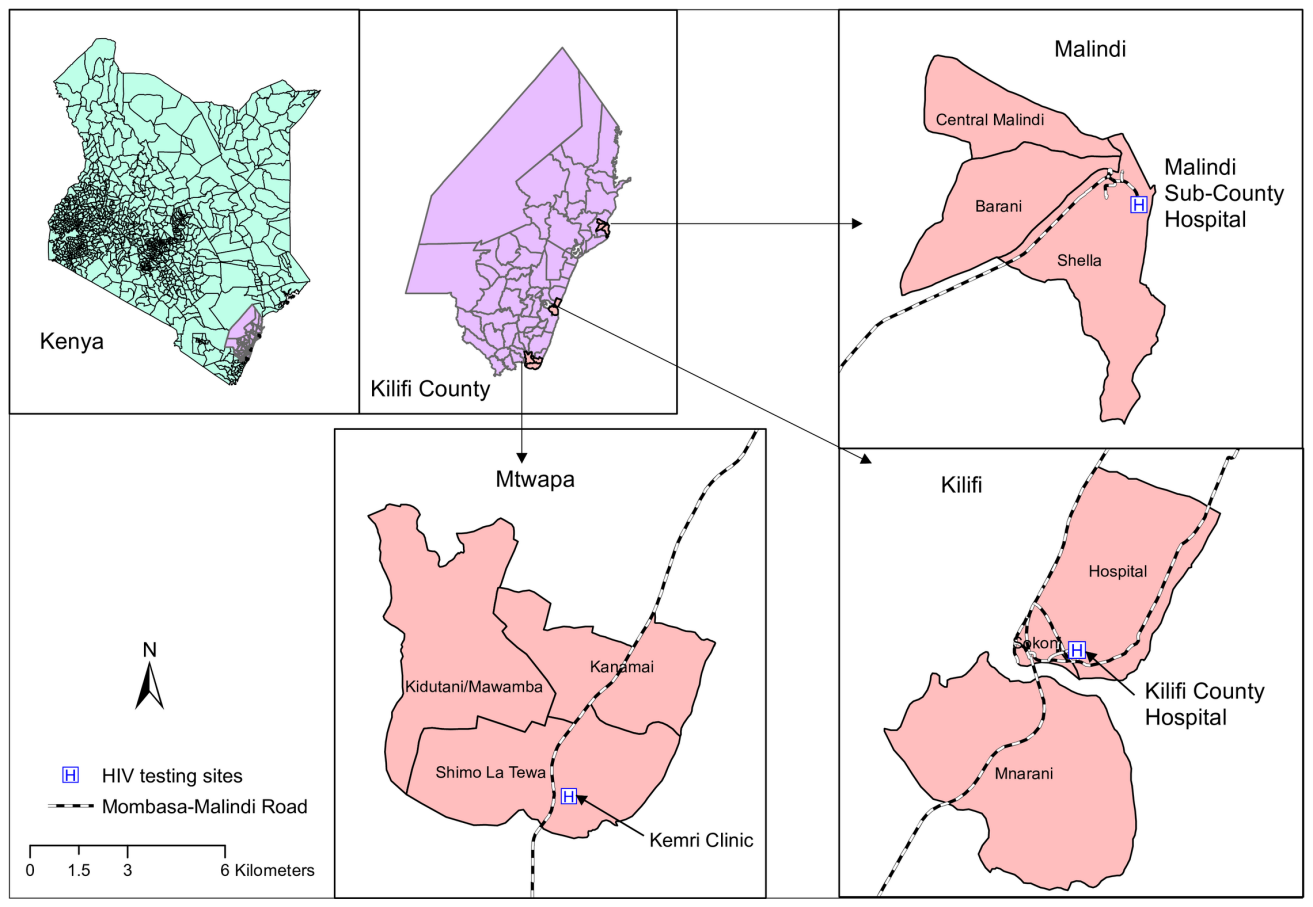
Map of study area.

Data were collected at three VCT centres operated by the Kenya Medical Research Institute (KEMRI)-Wellcome Trust Research Programme (KWTRP) in Kilifi county ( Figure 1, population 1.4 million ^20^). The three centres followed the serial testing strategy recommended in national guidelines ^14, 21^. The centres served clients seeking testing out of their own initiative (walk-in clients) and clients mobilized during periodic campaigns by KWTRP outreach workers (mobilized clients).

The oldest of these centres started operating in 2006 and is situated within the KWTRP main campus in Kilifi town, 60 kilometres (km) north of Mombasa (the second largest town in Kenya), and approximately 500 meters from the Kilifi County Hospital. The estimated catchment population for the county hospital is 125,500 ^22, 23^. HIV testing for the general population at the hospital started in 2004, and a large comprehensive HIV care centre was set up in 2005.

The second centre started operating in 2010 and is situated on the premises of the KWTRP clinic in Mtwapa town, 20 km north of Mombasa. Its estimated catchment population is 116,000 ^22, 23^. The town has a busy nightlife, with a large number of bars, nightclubs and hotels among other businesses, including many private health facilities and pharmacies ^24^. Since 2005, the KWTRP clinic has conducted cohort studies among KP, including MSM and FSW ^25^. The centre was set up following a request by community leaders who wanted clinic services to be accessible to the general population in the area.

The third centre also started operating in 2010 and is situated at a KWTRP-supported drop-in centre within the sub-county hospital in Malindi town, 120 km north of Mombasa. Its estimated catchment population is 128,000 ^22, 23^. This centre initially targeted MSM and FSW, but increasingly served the general population. During the period 2014–2015, KWTRP collaborated with community-based organizations to mobilize KP for testing.

### Data collection procedures

For each test encounter during the study period, a data collection form was completed by VCT staff capturing type of client (walk-in or mobilised in an outreach campaign), test location (Kilifi, Mtwapa, or Malindi), test date, gender, date of birth, highest level of education, religious affiliation, marital status, reason for testing, HIV test results, whether the client had ever tested before, and date of previous test (whether at our VCT or any other testing site). Starting in 2010, data were collected on self-reported HIV risk behaviour in the past six months, including: gender of sex partners, receipt of payment for sex, and current symptoms of sexually transmitted infections (STI). STI symptoms included: for men, urethral discharge and dysuria; for women, excessive or foul-smelling vaginal discharge; and for both men and women, genital sores and history of rectal discharge for those who reported anal sex. VCT records were extracted in early 2018, cleaned, and prepared for analysis.

### Sample selection

Our sample selection was guided by our goal to assess trends in adult walk-in VCT clients (i.e., clients seeking testing out of their own initiative). We therefore excluded data from mobilized clients who were tested during outreach campaigns and may have felt social pressure to test, even if previously diagnosed. In addition, the frequency and intensity of outreach campaigns varied over time, making it difficult to evaluate time trends. We also excluded VCT clients seeking confirmatory testing after a positive test done elsewhere, partners of HIV-positive index clients, Malindi clients from 2010–2011 (a period when testing exclusively targeted MSM), and clients outside the age group 18–39 years, where HIV incidence is highest in Kenya. We included 24,728 (52%) of all 47,893 test encounters in the original dataset ( *Extended data:* Supplementary Table 1). The dataset analysed included tests conducted at the Kilifi VCT centre in the period 2006–2017, Mtwapa VCT in 2010–2017, and Malindi VCT in 2012–2017.

### Data analysis

Data cleaning, recoding and analysis was conducted using Stata ^®^ version 15 (StataCorp, USA). Based on sex, sex of sex partners, and report of transactional sex (collected since 2010), we categorized clients into four risk groups: GP men, MSM, GP women, and FSW. As sexual behaviour data was not collected before 2010, test encounters from that period (all Kilifi-based) were categorized as GP.

The three main outcomes were HIV positivity (proportion of test encounters where the result was positive), proportion of encounters at which clients reported first-time testing (FTT), and proportion of encounters at which clients reported late retesting (previous test more than one year ago for GP or three months for KP). One year was defined as 365 calendar days, and three months as 90 days. We assessed change in outcomes over calendar year using locally weighted regression ( ^26^, Stata package “lowess”).

Using multivariable log binominal regression (“binreg”) and data from the period when information on sexual behaviour was complete (2012–2017, n=19,298), we assessed factors associated with the three outcomes. Given the difference in definitions of late retesting for GP and KP, we fit separate GP and KP models for this outcome. Age and sex were included a priori in all models; all other variables for which p<0.10 in bivariable analyses were carried forward in multivariable models. Factors with p<0.05 in the multivariable model were considered to have statistically significant associations with the outcome in question. For the FTT model, we assessed interactions between study area and risk group.

### Ethical statement

The study received ethical approval from the KEMRI Scientific and Ethical Review Unit (KEMRI/SERU/CGMR-C/188/4014).

## Results

### Characteristics of testing encounters

Of 24,728 tests conducted in the period 2006–2017, 50% were conducted in Mtwapa, 33% in Kilifi, and 16% in Malindi ( Table 1). Overall, 56% of encounters were among men, 68% among never-married individuals, 73% among Christians, and 41% among those with secondary education; 92% were among GP and 9% among KP; 32% were FTT encounters and 22% involved clients who were late retesters, that is, had a previous test more than a year ago for GP or three months for KP.

**Table 1.** Characteristics of HIV testing encounters among clients attending three voluntary counselling and testing centres in Kilifi County, Kenya, 2006–2017.

Characteristic	All centres combined		Mtwapa		Kilifi		Malindi	
	N	%	N	%	N	%	N	%
**Number of test** **encounters**	24,728	100	12,420	100	8,234	100	4,074	100
**Gender**								
Male	13,949	56	6,731	54	4,837	59	2,381	58
Female	10,779	44	5,689	46	3,397	41	1,693	42
**Age group**								
18–24 years	8,848	36	4,307	35	3,215	39	1,326	33
25–34 years	12,857	52	6,637	53	3,997	49	2,223	55
35–39 years	3,023	12	1,476	12	1,022	12	525	13
**Marital status ^1^**								
Never married	16,771	68	9,124	73	4,933	60	2,714	67
Married	6,594	27	2,810	23	2,766	34	1,018	25
Separated/ Divorced/ Widowed	1,361	6	486	4	533	6	342	8
**Religion**								
None	1,908	8	727	6	852	10	329	8
Christian	18,156	73	9,453	76	5,929	72	2,774	68
Muslim	4,664	19	2,240	18	1,453	18	971	24
**Education level**								
Primary or none	9,863	40	3,832	31	3,379	41	2,652	65
Secondary	10,064	41	5,934	48	3,061	37	1,069	26
Higher education	4,801	19	2,654	21	1,794	22	353	9
**Test period**								
2006–2009	2,357	10	0	0	2,357	29	0	0
2010–2011	3,636	15	1,781	14	1,855	23	0	0
2012–2014	8,219	33	4,741	38	2,063	25	1,415	35
2015–2017	10,516	42	5,898	48	1,959	24	2,659	65
**HIV testing** **history** ^2^								
On-time testing	11,291	46	7,140	57	2,956	36	1,195	29
Late retesting	5,563	22	3,127	25	1,513	18	923	23
First-time test	7,874	32	2,153	17	3,765	46	1,956	48
**Risk group** ^3^								
GP Men	12,502	51	6,268	50	4,729	57	1,505	37
MSM	1,447	6	463	4	108	1	876	22
GP Women	10,026	41	5,202	42	3,373	41	1,451	36
FSW	753	3	487	4	24	0	242	6
**Current STI** **symptoms** ^4^								
No	21,456	96	11,687	94	5,796	99	3,973	98
Yes	915	4	733	6	81	1	101	2

^1^ Data were missing for marital status (n=2) and risk group (n=1). ^2^ Late retesting was defined as previous test more than one year ago for GP or three months for key population. ^3^ Derived from gender of sex partners and report of transactional sex in past six months. These two variables were collected from 2010 onwards. All test encounters before 2010 (all Kilifi-based) were categorized as GP. ^4^ Any report of urethritis, dysuria, vaginal discharge, genital sore, or proctitis. As these variables were only collected from 2010 onwards, data were missing for 2,603 test encounters. *GP: General population; MSM: Men who have Sex with Men; FSW: Female Sex Workers; STI: Sexually Transmitted Infection*

### Time trends in the proportion of encounters with first-time testers

**Figure 2.**
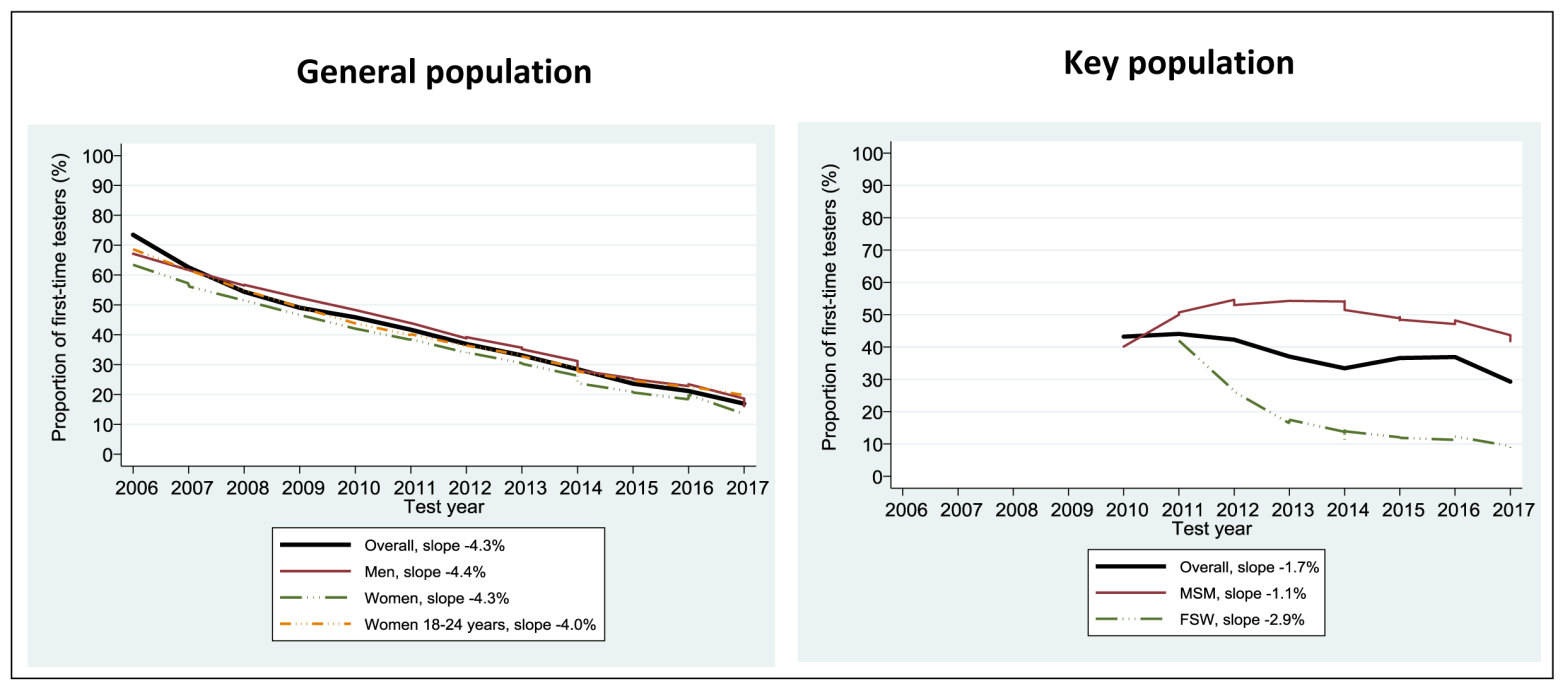
Time trends in the proportion of encounters involving first-time testers among clients attending voluntary counselling and testing centres in Kilifi County, Kenya. *Plots drawn using locally weighted scatterplot smoothing (LOWESS). Slope is percentage-point change per year. Data points on which the plots are based are included in the supplemental materials (Extended data: Supplementary Table 2 and 3). MSM: Men who have Sex with Men; FSW: Female Sex Workers.*

For GP, we observed a decline in the proportion of encounters where the client was testing for the first time among men overall, women overall, and women aged 18–24 years ( Figure 2). Slopes were similar for all three sub-groups. For KP, the proportion of encounters that involved FTT declined less steadily, with the lowest percentage-point decline per year observed in MSM.

For the final year assessed (2017), the proportion of encounters involving FTT was 15% for GP clients: 16% for men, 13% for women, and 20% for women aged 18–24 years. The proportion of encounters involving FTT was 29% for KP: 42% for MSM and 9% for FSW.

### Time trends in the proportion of encounters with late retesters

**Figure 3.**
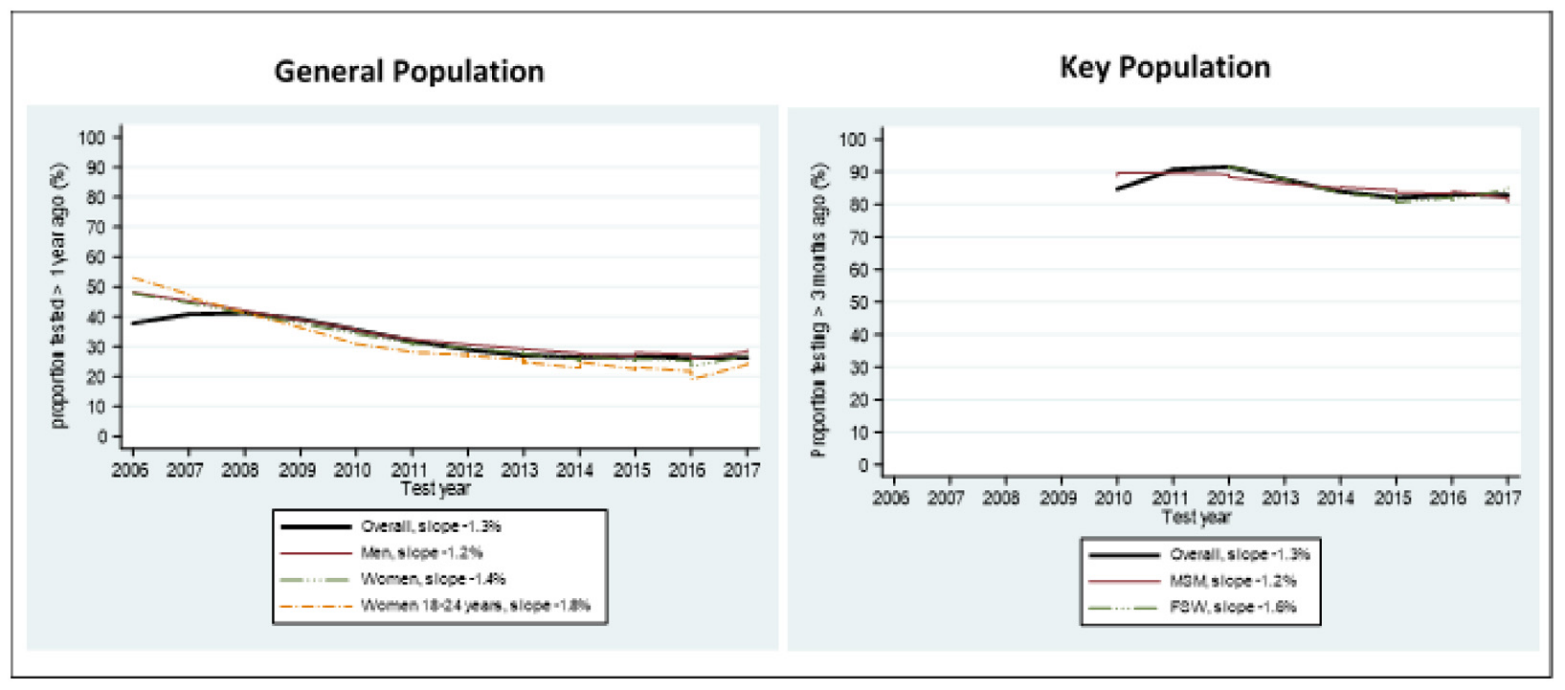
Time trends in the proportion of encounters involving late retesting among clients attending voluntary counselling and testing centers in Kilifi County, Kenya. *Plots drawn using locally weighted scatterplot smoothing (LOWESS). Slope is percentage-point change per year. (Extended data: Supplementary Table 4 and 5). MSM: men who have sex with men; FSW: female sex workers.*

We observed declines in the proportion of encounters involving late retesting for both GP (previous test more than one year ago) and KP (previous test more than three months ago) ( Figure 3). Throughout the period assessed, the proportion of encounters involving late retesting among the KP remained much higher than that in GP. The percentage-point changes per year were similar for all sub-groups assessed.

For the final year assessed (2017), the proportion of encounters involving late-retesting was 28% for GP: 29% for encounters with men, 28% for encounters with women, and 25% for encounters with women aged 18–24 years. The proportion of encounters involving late retesting was 83% for KP: 81% for MSM encounters and 85% for FSW encounters.

### Time trends in HIV positivity

**Figure 4.**
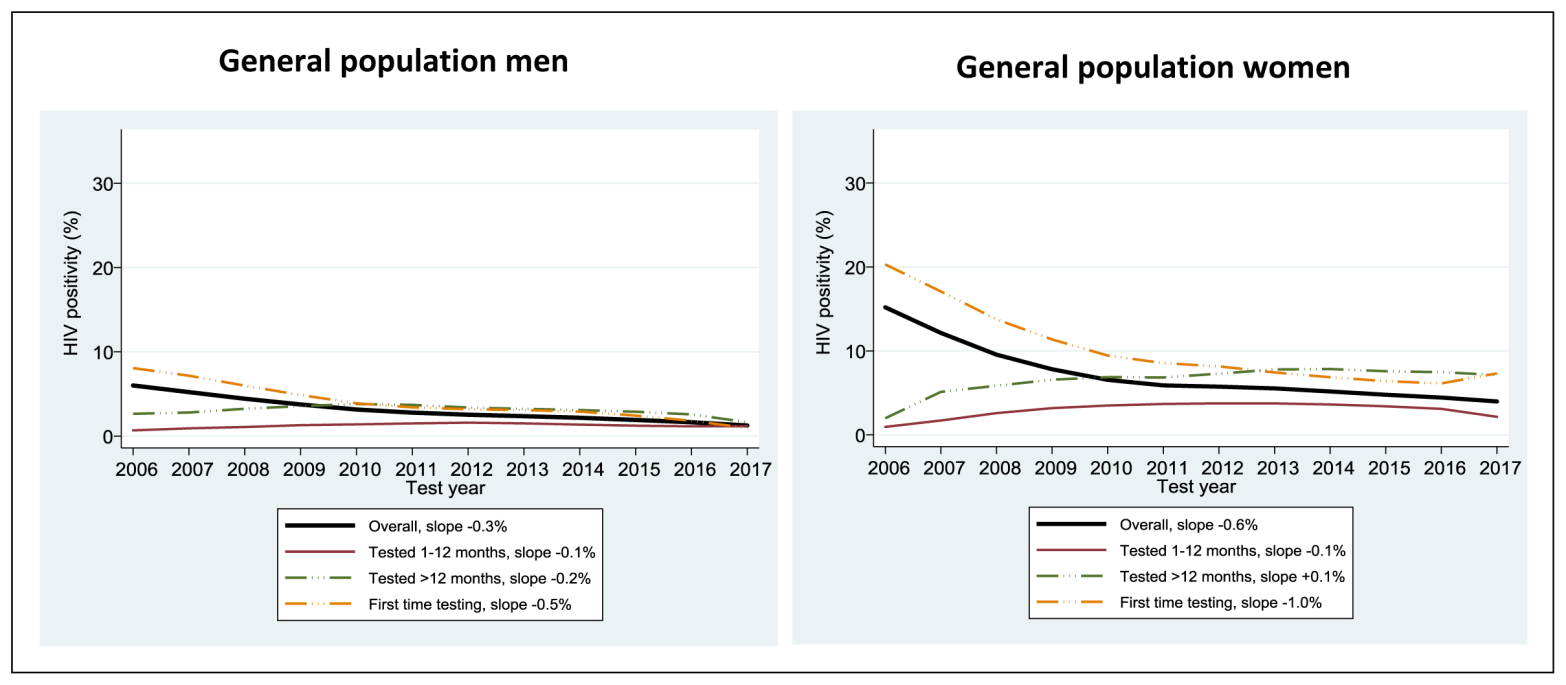
Time trends in HIV positivity at testing encounters among general population clients attending voluntary counselling and testing centres in Kilifi County, Kenya. *Plots drawn using locally weighted scatterplot smoothing (LOWESS). (Extended data: Supplementary Table 6 and 7).*

For GP, there was a decline in overall HIV positivity at encounters with both men and women, as well as with the sub-group of those testing for the first time, but not among female late re-testers ( Figure 4).

**Figure 5.**
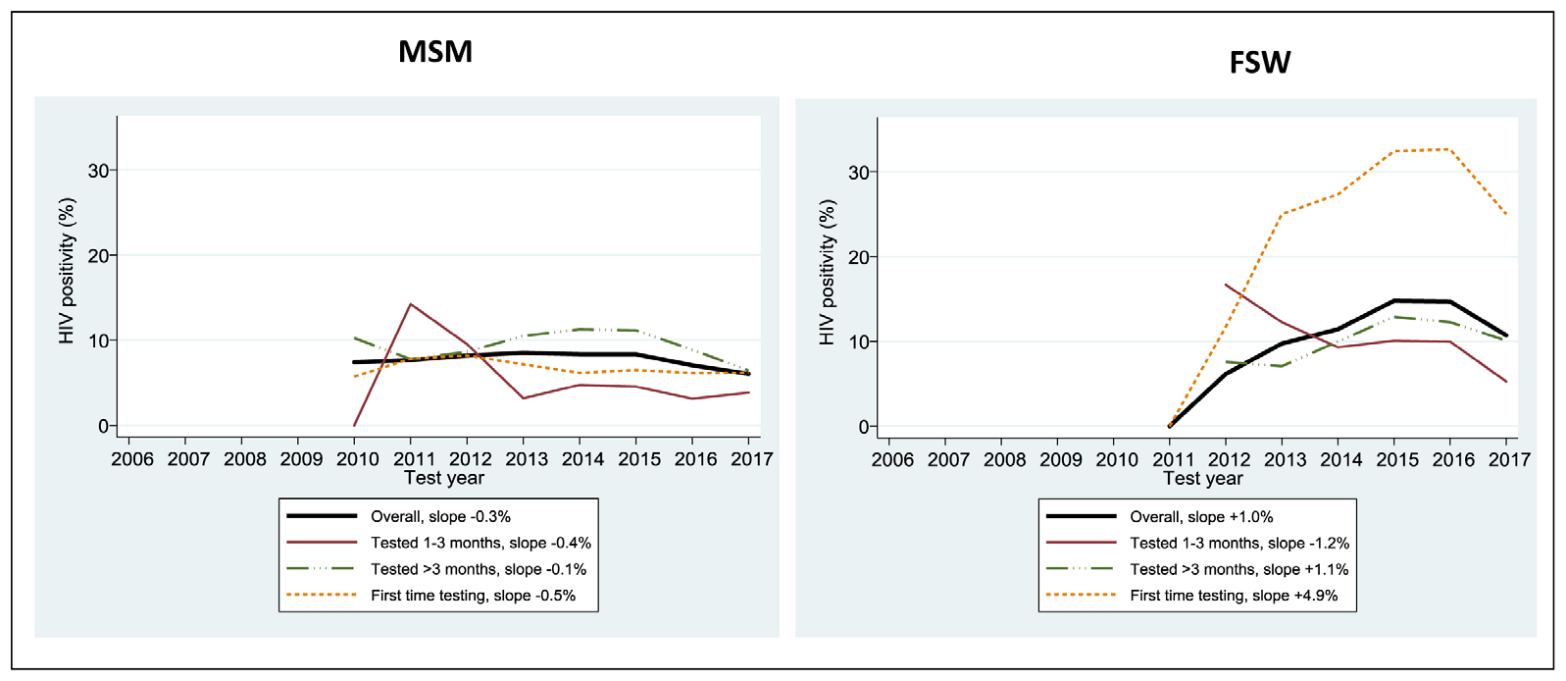
Time trends in HIV positivity at testing encounters among key population clients attending voluntary counselling and testing centres in Kilifi County, Kenya. *Plots drawn using locally weighted scatterplot smoothing (LOWESS). Slope is percentage-point change per year. (Extended data: Supplementary Table 8 and 9).*

For encounters among MSM, HIV positivity was steady among encounters involving late re-testing ( Figure 5). For encounters among FSW, there was an increase in HIV positivity over time. This was also true for encounters with FSW involving first-time testing (large increase at +4.9 percentage points per year) and late re-testing, but not for encounters with FSW involving on-time re-testing.

For the final year assessed (2017), overall HIV positivity for GP encounters was 2.3%: 1.1% for encounters with men, 3.9% for encounters with women, and 2.8% for encounters with women aged 18–24 years. Overall HIV positivity in KP encounters was 7.8%: 6.0% for MSM encounters and 10.7% for FSW encounters.

### Factors associated with first-time testing encounters

As presented in Table 2, factors associated with increased probability of FTT at VCT encounters included: test location (Kilifi and Malindi), age 18–24 years, never-married status, lower educational attainment. Compared to GP women encounters, MSM and GP men encounters were more likely to involve FTT, while FSW encounters were less likely to involve FTT. First-time testing encounters were less likely during earlier testing periods and among clients with current STI symptoms. No interactions between study area and risk group were identified (data not shown).

**Table 2.** Factors associated with first-time testing encounters among adult clients attending voluntary counselling and testing centres in Kilifi, Kenya, 2012–2017.

Factor	Number of test encounters ^1^	Number (%) first- time testing encounters	Bivariable analysis		Multivariable analysis (Full model)	
			Risk ratio [95% Confidence interval]	P value	Adjusted risk ratio [95% Confidence interval]	P value
**Testing location**						
Kilifi	4,022	1,355 (27.2)	2.2 [2.0-2.3]	<0.001	2.1 [1.9-2.2]	<0.001
Malindi	4,074	1,956 (39.3)	3.1 [2.9-3.2]	<0.001	2.7 [2.5-2.9]	<0.001
Mtwapa	10,639	1,666 (33.5)	Ref	Ref	Ref	Ref
**Age group**
18–24 years	6,440	2,031 (40.8)	1.4 [1.3-1.5]	<0.001	1.3 [1.2-1.4]	<0.001
25–34 years	9,969	2,415 (48.5)	1.1 [1.0-1.2]	0.158	1.0 [0.9-1.1]	0.509
35–39 years	2,326	531 (10.7)	Ref	Ref	Ref	Ref
**Marital status ^2^**						
Never married	13,026	3,622 (72.8)	1.2 [1.1-1.2]	<0.001	1.2 [1.1-1.3]	<0.001
Separated/ Divorced/ Widowed	937	218 (4.4)	1.0 [0.9-1.1]	0.708	0.9 [0.8-1.1]	0.236
Married	4,770	1,137 (22.8)	Ref	Ref	Ref	Ref
**Religion**						
None	1,308	364 (7.3)	1.1 [1.0-1.2]	0.078	1.0 [0.9-1.1]	0.959
Muslim	3,560	1,060 (21.3)	1.2 [1.1-1.2]	<0.001	1.0 [0.9-1.1]	0.707
Christian	13,867	3,553 (71.4)	Ref	Ref	Ref	Ref
**Education level**						
Primary or none	7,271	2,480 (49.8)	1.8 [1.7-2.0]	<0.001	1.6 [1.4-1.7]	<0.001
Secondary	7,727	1,794 (36.0)	1.2 [1.1-1.3]	<0.001	1.3 [1.2-1.4]	<0.001
Higher Education	3,737	703 (14.1)	Ref	Ref	Ref	Ref
**Testing period**						
2015–2017	10,516	2,428 (48.8)	0.7 [0.7-0.8]	<0.001	0.7 [0.7-0.7]	<0.001
2012–2014	8,219	2,549 (51.2)	Ref	Ref	Ref	Ref
**Risk group** ^3^						
GP men	9,262	2,515 (50.5)	1.2 [1.1-1.3]	<0.001	1.3 [1.2-1.4]	<0.001
MSM	1,256	640 (12.9)	2.2 [2.1-2.4]	<0.001	1.4 [1.3-1.6]	<0.001
FSW	751	112 (2.3)	0.7 [0.5-0.8]	<0.001	0.6 [0.5-0.7]	<0.001
GP women	7,466	1,710 (34.4)	Ref	Ref	Ref	Ref
**Current STI symptoms**						
Yes	848	146 (2.9)	0.6 [0.5-0.7]	<0.001	0.7 [0.6-0.9]	<0.001
No	17,887	4,831 (97.1)	Ref	Ref	Ref	Ref

^1^ For Mtwapa and Kilifi, numbers are less than in Table 1 since the time periods are different. ^2^ Data were missing for marital status (n=2). ^3^ Gender and transactional sex were excluded from the model due to collinearity with the risk group variable. *GP: General population; MSM: Men who have sex with men; FSW: Female sex workers; STI: Sexually transmitted infection.*

### Factors associated with late retesting encounters

The GP model is presented in Table 3, and the KP model in *Extended data:* Supplementary Table 10. The KP model did not identify any predictors of late retesting (previous test more than three months ago).

**Table 3.** Factors associated with late retesting encounters among general population clients attending voluntary counselling and testing centres in Kilifi, Kenya, 2012–2017.

Factor	Number of test encounters ^1^	Numbe (%) of late- retesting encounters	Bivariable analysis		Multivariable analysis (Full model)	
			Risk ratio [95% Confidence interval]	P value	Adjusted risk ratio [95% Confidence interval]	P value
**Testing location**						
Kilifi	2,612	753 (22.7)	1.1 [1.1-1.2]	0.001	1.1 [1.0-1.2]	0.039
Malindi	1,603	456 (13.7)	1.1 [1.0-1.2]	0.012	1.1 [1.0-1.2]	0.079
Mtwapa	8,288	2,112 (63.6)	Ref	Ref	Ref	Ref
**Age group**						
18–24 years	3,906	971 (29.2)	0.8 [0.7-0.9]	<0.001	0.9 [0.8-1.0]	0.065
25–34 years	6,897	1,831 (55.1)	0.9 [0.8-0.9]	0.001	0.9 [0.8-1.0]	0.081
35–39 years	1,700	519 (15.6)	Ref	Ref	Ref	Ref
**Marital status**						
Never married	8,367	2,105 (63.4)	0.9 [0.8-0.9]	<0.001	0.9 [0.8-1.0]	0.009
Separated / Divorced/ Widowed	626	202 (6.1)	1.1 [1.0-1.3]	0.084	1.1 [1.0-1.3]	0.157
Married	3,508	1,014 (30.5)	Ref	Ref	Ref	Ref
**Religion**						
None	849	195 (5.9)	0.8 [0.7-1.0]	0.007	0.8 [0.7-1.0]	0.012
Muslim	2,189	532 (16.0)	0.9 [0.8-1.0]	0.004	0.9 [0.8-1.0]	0.010
Christian	9,465	2,594 (78.1)	Ref	Ref	Ref	Ref
**Education level**						
Primary or none	4,196	1,147 (34.5)	1.0 [0.9-1.0]	0.363	1.0 [0.9-1.1]	0.487
Secondary	5,390	1,348 (40.6)	0.9 [0.8-1.0]	0.001	0.9 [0.8-1.0]	0.024
Higher Education	2,917	826 (24.9)	Ref	Ref	Ref	Ref
**Testing period**						
2015–2017	7,390	1,891 (56.9)	0.9 [0.9-1.0]	0.003	0.9 [0.8-1.0]	0.006
2012–2014	5,113	1,430 (43.1)	Ref	Ref	Ref	Ref
**Risk group**						
GP men	6,747	1,841 (55.4)	1.1 [1.0-1.1]	0.047	1.1 [1.0-1.1]	0.069
GP women	5,756	1,480 (44.6)	Ref	Ref	Ref	Ref
**Current STI symptoms**						
Yes	508	105 (3.2)	0.8 [0.6-0.9]	0.003	0.8 [0.7-1.0]	0.019
No	11,995	3,216 (96.8)	Ref	Ref	Ref	Ref

^1^ For Mtwapa and Kilifi, numbers are less than in Table 1 since the time periods are different. *GP: General population; STI: Sexually transmitted infection*.

In the GP model, encounters involving late retesting (previous test more than one year ago) were less likely among never-married clients, clients professing Muslim or no religious affiliation, those with secondary education, and those with current STI symptoms. Encounters involving late retesting were more likely during 2012–2014 and among clients served in Kilifi compared to Mtwapa.

### Factors associated with HIV positivity

Table 4 presents factors associated with HIV positivity at testing encounters. Encounters with a positive test result were more likely to involve Malindi clients, clients with less than higher education, first-time testers and late re-testers, MSM and FSW, and clients with current STI symptoms. Of note, encounters involving FTT and late retesting were about twice as likely to result in a positive test compared to on-time retesting encounters. Encounters among clients with primary or no education were 1.6 times more likely to result in a positive HIV test, compared to those among clients with higher education, while those among clients with secondary education were 1.5 times more likely.

**Table 4.** Factors associated with HIV positivity at testing encounters among adult clients attending voluntary counselling and testing centres in Kilifi, Kenya, 2012–2017.

Factor	Number tested	Number (%) positive	Bivariable analysis		Multivariable analysis (Full model)	
			Risk ratio [95% Confidence interval]	P value	Adjusted risk ratio [95% Confidence interval]	P value
**Testing location**						
Kilifi	4,022	122 (16.8)	0.9 [0.8-1.2]	0.576	0.9 [0.7-1.1]	0.223
Malindi	4,074	262 (36.1)	2.0 [1.7-2.3]	<0.001	1.3 [1.1-1.5]	0.011
Mtwapa	10,639	342 (47.1)	Ref	Ref	Ref	Ref
**Age group**						
18–24 years	6,440	168 (23.1)	0.4 [0.4-0.5]	<0.001	0.5 [0.4-0.6]	<0.001
25–34 years	9,969	420 (57.9)	0.7 [0.6-0.9]	<0.001	0.8 [0.6-0.9]	0.008
35–39 years	2,326	138 (19.0)	Ref	Ref	Ref	Ref
**Marital status**						
Never married	13,026	382 (52.6)	0.6 [0.5-0.6]	<0.001	0.6 [0.5-0.7]	<0.001
Separated/ Divorced/ Widowed	937	92 (12.7)	1.9 [1.5-2.3]	<0.001	1.2 [1.0-1.5]	0.081
Married	4,770	252 (34.7)	Ref	Ref	Ref	Ref
**Religion**						
None	1,308	63 (8.7)	1.3 [1.0-1.6]	0.062	1.3 [1.0-1.6]	0.067
Muslim	3,560	139 (19.1)	1.0 [0.9-1.2]	0.726	0.9 [0.8-1.1]	0.578
Christian	13,867	524 (72.2)	Ref	Ref	Ref	Ref
**Education level**						
Primary/none	7,271	382 (52.6)	2.7 [2.1-3.5]	<0.001	1.6 [1.3-2.1]	<0.001
Secondary	7,727	272 (37.5)	1.8 [1.4-2.4]	<0.001	1.5 [1.2-1.9]	0.002
Higher Education	3,737	72 (9.9)	Ref	Ref	Ref	Ref
**Testing period**						
2015–2017	10,516	371 (51.1)	0.8 [0.7-0.9]	0.005	0.8 [0.7-0.9]	0.007
2012–2014	8,219	355 (48.9)	Ref	Ref	Ref	Ref
**HIV testing history ^1^**						
On-time testing	9,375	212 (29.2)	Ref	Ref	Ref	Ref
Late retesting	4,383	260 (35.8)	2.6 [2.2-3.1]	<0.001	2.0 [1.6-2.4]	<0.001
First-time testing	4,977	254 (35.0)	2.3 [1.9-2.7]	<0.001	2.0 [1.7-2.5]	<0.001
**Risk group ^2^**						
MSM	1,256	99 (13.6)	1.6 [1.3-2.0]	<0.001	1.1 [0.9-1.4]	0.341
FSW	751	90 (12.4)	2.5 [2.0-3.1]	<0.001	1.8 [1.4-2.3]	<0.001
GP men	9,262	176 (24.2)	0.4 [0.3-0.5]	<0.001	0.4 [0.3-0.5]	<0.001
GP Women	7,466	361 (49.7)	Ref	Ref	Ref	Ref
**Current STI symptoms**						
Yes	848	59 (8.1)	1.9 [1.4-2.4]	<0.001	1.6 [1.2-2.0]	0.001
No	17,887	667 (91.9)	Ref	Ref	Ref	Ref

^1^Late retesting was defined as previous test more than one year ago for GP or three months for key population. ^2^Gender and transactional sex were excluded from the model due to collinearity with the risk group variable. *GP: General population; MSM: Men who have sex with men; FSW: Female sex workers; STI: Sexually transmitted infection.*

Encounters where a positive HIV result was less likely were among clients under 35 years, those who were never married, and GP men.

## Discussion

Analysis of 12-year data from three VCT centres in Kilifi county, Kenya, revealed a decline in the proportion of encounters involving first-time testing (those who had never tested before) among GP men, GP women, GP women aged 18–24 years, and FSW; suggesting increasing coverage of HIV testing in the county, in line with national trends ^6^. However, the proportion of encounters involving FTT among MSM was relatively constant, and the prevalence of FTT encounters among MSM in the final year assessed (2017) was relatively high at 42% (compared to 15% in GP and 9% in FSW). We also found an overall decline – albeit more modest – in the proportion of encounters involving late retesting, but this remained, in absolute terms, much higher among KP, for whom more frequent testing is recommended, compared to GP. While the proportion of encounters involving late retesting (i.e. previous test more than a year ago) was 28% for GP in 2017, 83% of encounters for KP in the current study involved late retesting (previous test more than 3 months ago).

FTT encounters were more common among men (both GP men and MSM), among younger (18–24 years) or single persons, and among persons with lower educational attainment. Such persons may perceive themselves to be at higher risk. Among GP, late retesting encounters were less common among single persons, those with secondary education, those professing Muslim or no religious affiliation and those who had current STI symptoms. While the association with religious affiliation is less clear, the other associations may indicate increased awareness of risk for HIV. These findings are of interest given that encounters involving first-time and late re-testing were more likely to yield a positive test result, compared to on-time re-testers. Increased education and a mobilization strategy targeting sub-groups with these attributes could potentially contribute significantly to achieving the 90% UNAIDS HIV diagnosis target in Kilifi county and other similar settings. Because current STI symptoms were associated with a near doubling of HIV positivity at encounters, such approaches should incorporate integrated sexual reproductive health services that include screening, diagnosis and treatment for STI ^27^.

Encounters in which HIV test results were positive declined among GP men and women, and among MSM, but increased among FSW. The increase in positivity among FSW may have been due to a high proportion of encounters involving FTT among FSW in one location (Malindi, data not shown) as a result of increased knowledge or risk perception after cumulative outreach efforts in recent years. While MSM-focused community-based organizations were active in the three areas from the beginning of the study period, there were initially limited services targeting FSW ^28^. At the KWTRP VCT centre in Malindi, specifically, the initial focus was on MSM and did not expand to include FSW until 2015 ^29^.

The low uptake of quarterly retesting (implied by the low proportion of on-time retesting encounters among KP in our study) and consequent continuing transmission among MSM and FSW may be due to stigmatizing attitudes of healthcare workers, discrimination, and concerns about confidentiality; factors that have been shown to decrease access to health services ^30– 32^ HIV self-testing (HIVST) services were introduced in Kenya in 2017 in order to improve test uptake among hard-to-reach populations including KP, men and young people ^33^. Scaling up of HIVST and partner notification services among KP, including through innovative strategies such as peer test distribution, has been shown to increase test uptake in this population ^34– 37^.

In our study, low educational attainment was associated not only with encounters for FTT but also with HIV positivity. Testing encounters among clients with primary or no education were 1.6 times more likely to result in a positive HIV test, compared to those among clients with higher education, while those among clients with secondary education had 1.5 times the likelihood. Kilifi County is amongst the poorest counties in the country ^17^, and has low literacy levels ^18, 19^. Specifically, in Kilifi county, educational outreach and targeted HIV testing programs tailored to the needs of low-literacy, rural populations might improve HIV testing services. For instance, HIV knowledge and literacy could be assessed among patients seeking healthcare, and patients with no or low level of education could be offered brief education sessions with visual aids and confidential HIV testing with clear and simple messages. Community outreach could also help to dispel myths about HIV and increase awareness and uptake of services.

In Kenya and other similar settings, adolescents and young women 15–24 years are disproportionately affected by HIV ^38^. In 2017, this sub-population accounted for more than a third of all new adult HIV infections in Kenya ^5, 6^; HIV prevalence in this group was estimated at 2.6% ^5, 6^. In the present study, HIV positivity at VCT encounters by young women 18–24 years was 2.8% in 2017. Initiatives that tackle social determinants of HIV risk in this vulnerable group, such as poverty, gender inequality, and sexual violence are needed ^39– 41^. However, resources to implement such initiatives may be limited, since Kilifi is categorized as a medium priority county for HIV prevention and care. Less donor-dependent interventions, such as sex education at primary and secondary schools, will be crucial and could be rolled out in tandem with HIV education aimed at improving health literacy.

Our findings suggest unequal delivery of HIV prevention services across the county. Testing history and HIV positivity at VCT encounters varied by town, with Malindi having the lowest testing prevalence and highest HIV positivity. Malindi is more geographically isolated, being furthest from Mombasa – Kenya’s second largest city, main seaport and former administrative headquarters for the coast province. On the other hand, the town has a vibrant tourism sector which attracts large numbers of KP. Clearly, greater coverage of HIV testing and prevention services is needed in this area, with a strong focus on KP.

This study demonstrates the utility of rigorous analysis of routinely collected data to evaluate trends in first-time testing, late retesting, and HIV positivity at VCT encounters at a county level ^42^. Currently, test data collected at various testing facilities are reported to county headquarters only in summary form, combining data from VCT centres and other testing points such as provider-initiated testing in outpatient and antenatal clinics; the data is also not disaggregated by risk groups. Our findings also show that additional socio-demographic, sexual behaviour, and testing history data can be useful in identifying sub-populations in need of additional education and outreach, as well as targeted HIV prevention and care services.

Our study had a number of limitations. First, we cannot be sure that encounters with a positive test result documented were new diagnoses of HIV infection, as stigma and social desirability bias may lead some clients to report their previous test result as negative even if it was positive ^43, 44^. Second, social desirability bias may also have resulted in over-reporting of previous HIV test uptake. Third, stigma and discrimination towards MSM may have resulted in under-reporting of same-sex behavior practices among men and sex work stigma may have resulted in under-reporting of transactional sex among women. Additionally, our dataset lacked information on sexual behaviour prior to 2010, limiting our ability to describe trends by risk group in that period. Fourth, the data capture system we used did not track individual testers longitudinally, precluding our ability to analyse individual testing practices over time. As one individual’s multiple retesting episodes were counted as individual encounters, this may have biased our modelling. Fifth, cross-site comparisons of time trends may have been biased by changes in covered populations in the different VCT centers over time. Sixth, although we excluded clients mobilized through outreach activities, some of the clients registered as walk-in may have been influenced indirectly by outreach activities, hence the sample used may not be wholly representative of the walk-in VCT clientele. Seventh, our data do not enable us to hypothesize about mechanisms underlying some findings, such as associations with religion, and some findings may be due to chance or residual confounding. In particular, the use of p values to select variables for model building can be misleading ^45^. Finally, the three VCT centres included in the study are close to KWTRP research clinics, hence clients may not be representative of the whole VCT clientele in the county.

## Conclusions

Our study showed that in Kilifi county, HIV positivity at encounters in the three VCT centres studied was most common when encounters involved first-time testing, testing less than annually, key populations, and persons with lower educational attainment. While encounters involving first-time testing and late retesting decreased over time, potentially reflecting increased testing coverage, there is an urgent need to evaluate actual HIV test coverage in different sub-populations and to implement non-stigmatizing HIV testing programs accessible to all in order to achieve the 90% diagnosis target set for the county.

## Data availability

### Underlying data

Harvard Dataverse: Underlying dataset for: Trends and predictors of HIV positivity and time since last test at voluntary testing and counseling encounters among adults in Kilifi, Kenya, 2006–2017, https://doi.org/10.7910/DVN/43DAWU ^46^.

This project contains the following underlying data:

- Underlying data
- Codebook

### Extended data

Harvard Dataverse: Supplementary tables for: Trends and predictors of HIV positivity and time since last test at voluntary testing and counseling encounters among adults in Kilifi, Kenya, 2006–2017, https://doi.org/10.7910/DVN/TVQJZP ^47^.

This project contains the following extended data:

- Supplementary Table 1. Number of test encounters excluded from the analysis and HIV positivity for each exclusion category.
- Supplementary Table 2. Time trends in the proportion of encounters involving first-time testers among general population attending voluntary counselling and testing centres in Kilifi County, Kenya, N = 22,528
- Supplementary Table 3 Time trends in the proportion of encounters involving first-time testers among key population attending voluntary counselling and testing centres in Kilifi County, Kenya, N = 2,200
- Supplementary Table 4. Time trends in the proportion of encounters involving late retesting among general population attending voluntary counselling and testing centers in Kilifi County, Kenya, N = 15,488
- Supplementary Table 5. Time trends in the proportion of encounters involving late retesting among key population attending voluntary counselling and testing centers in Kilifi County, Kenya, N = 1,366
- Supplementary Table 6. Time trends in HIV positivity at testing encounters among general population men attending voluntary counselling and testing centres in Kilifi County, Kenya, N = 12,502
- Supplementary Table 7. Time trends in HIV positivity at testing encounters among general population women attending voluntary counselling and testing centres in Kilifi County, Kenya, N = 10,026
- Supplementary Table 8. Time trends in HIV positivity at testing encounters among MSM attending voluntary counselling and testing centres in Kilifi County, Kenya, N = 1,447
- Supplementary Table 9. Time trends in HIV positivity at testing encounters among FSW attending voluntary counselling and testing centres in Kilifi County, Kenya, N = 729
- Supplementary Table 10. Factors associated with testing less than quarterly (> 3 months ago) among episodes with Key Populations, voluntary counselling and testing centres in Kilifi County, Kenya, 2012–2017

Data are available under the terms of the Creative Commons Zero "No rights reserved" data waiver (CC0 1.0 Public domain dedication).
