# Supplementary material for: Trends and predictors of HIV positivity and time since last test at voluntary counselling and testing encounters among adults in Kilifi, Kenya, 2006-2017
Source: Wellcome Open Res. 2021 Jun 4;4:127. Originally published 2019 Aug 23. [Version 3] doi: 10.12688/wellcomeopenres.15401.3 (PMC8042516; doi:10.12688/wellcomeopenres.15401.3)
Supplement: Peer review history

# Peer review: Reviewer response for version 2

**DOI:** 10.21956/wellcomeopenres.17809.r42696

**Published:** 2021-04-12

**Authors:** Mathieu Maheu-Giroux

**Affiliations:** 1 Department of Epidemiology, Biostatistics, and Occupational Health, School of Global and Population Health, McGill University, Montreal, Canada

**Comments**

1. Within Figure 2 and 3 captions, it is written: "Due to the small number of FSW clients from Kilifi location, data for 24 FSW were not included in the figure." It is unclear to us why those participants were excluded from those figures since a small number of participants is not a sufficient reason for exclusion. Those participants must be included in the analyses and if not possible, exact and relevant reasons for exclusion should be explained in the Data analysis section of the manuscript.
2. In their answer to our previous comment saying that inclusion of variables based on their statistical significance could be misleading, we don't understand why the authors said: "The issue of variables being excluded because of p>0.1 in bivariable analysis that could have had P<0.1 in adjusted analysis does not arise for our study since, as explained here above, all variables were included in the multivariable model. [Pages 8, (data analysis)]". Indeed, in the Data analysis section of the manuscript, it is rather clearly said that "Variables with p<0.10 from bivariable analyses were included in the multivariable model", which suggest that variables with p>0.1 in bivariable analyses were excluded from multivariable models and thus that not all variables were included in the multivariable model.
3. Figures 4 and 5: the Y axis title should be "HIV positivity" and not "Proportion newly diagnosed".
4. To support the following statement in the Discussion section: "First, we cannot be sure that encounters with a positive test result documented were new diagnoses of HIV infection, as stigma and social desirability bias may lead some clients to report their previous test result as negative even if it was positive." we suggest to reference a meta-analysis of misreporting of known HIV status (Soni et al. 2020 ^1^. Under-reporting of known HIV-positive status among people living with HIV: a systematic review and meta-analysis) that can be found here as a preprint.
5. Please correct the following typo: "literacacy" Page 9.

Is the work clearly and accurately presented and does it cite the current literature?

Partly

If applicable, is the statistical analysis and its interpretation appropriate?

No

Are all the source data underlying the results available to ensure full reproducibility?

Yes

Is the study design appropriate and is the work technically sound?

Yes

Are the conclusions drawn adequately supported by the results?

No

Are sufficient details of methods and analysis provided to allow replication by others?

Partly

Reviewer Expertise:

Infectious disease epidemiology; HIV and other sexually transmitted and blood-borne infections; Impact Evaluation; Mathematical modeling; Population health

I confirm that I have read this submission and believe that I have an appropriate level of expertise to confirm that it is of an acceptable scientific standard.

## References

[1] : Under-Reporting of Known HIV-Positive Status Among People Living with HIV: A Systematic Review and Meta-Analysis. *SSRN Electronic Journal* .2020; 10.2139/ssrn.3690926 10.2139/ssrn.3690926 PMC860223334046763

# Response

**Published:** 2021-05-03

**Authors:** Peter Mugo

**Affiliations:** Kenya Medical Research Institute, Kenya

**Within Figure 2 and 3 captions, it is written: "Due to the small number of FSW clients from Kilifi location, data for 24 FSW were not included in the figure." It is unclear to us why those participants were excluded from those figures since a small number of participants is not a sufficient reason for exclusion. Those participants must be included in the analyses and if not possible, exact and relevant reasons for exclusion should be explained in the Data analysis section of the manuscript.**

*We thank the reviewer for pointing this out. This exclusion was a carry-over from a previous version of the graphs that included site-specific panels by population group: the small number of FSWs in Kilifi was causing wild ups and downs. We have revised the graph to include them and removed the relevant text from the figure captions.*

**In their answer to our previous comment saying that inclusion of variables based on their statistical significance could be misleading, we don't understand why the authors said: "The issue of variables being excluded because of p>0.1 in bivariable analysis that could have had P<0.1 in adjusted analysis does not arise for our study since, as explained here above, all variables were included in the multivariable model. [Pages 8, (data analysis)]". Indeed, in the Data analysis section of the manuscript, it is rather clearly said that "Variables with p<0.10 from bivariable analyses were included in the multivariable model", which suggest that variables with p>0.1 in bivariable analyses were excluded from multivariable models and thus that not all variables were included in the multivariable model.**

*Age and sex were included a priori, and other variables were indeed carried forward if the p value from bivariable analysis was <-10. We have clarified this in our revised manuscript. We agree with the reviewer that inclusion of variables based on their statistical significance alone can lead to misleading conclusions, and have added this as a limitation of our study.*

**Figures 4 and 5: the Y axis title should be "HIV positivity" and not "Proportion newly diagnosed".**

*We have corrected this error.*

**To support the following statement in the Discussion section: "First, we cannot be sure that encounters with a positive test result documented were new diagnoses of HIV infection, as stigma and social desirability bias may lead some clients to report their previous test result as negative even if it was positive." we suggest to reference a meta-analysis of misreporting of known HIV status (Soni et al. 20201. Under-reporting of known HIV-positive status among people living with HIV: a systematic review and meta-analysis) that can be found here as a preprint.**

*We thank the reviewer for bringing this to our attention. We have added the suggested reference.*

**Please correct the following typo: "literacacy" Page 9.**

*We have corrected the typo. Thanks.*

# Peer review: Reviewer response for version 1

**DOI:** 10.21956/wellcomeopenres.16834.r39379

**Published:** 2020-07-15

**Authors:** Parinita Bhattacharjee

**Affiliations:** 1 Centre for Global Public Health, University of Manitoba, Winnipeg, Canada

The subject of the paper is very important. As the country is debating the need for retesting or frequent testing, this paper would be an important resource. 

 The paper is being reviewed and possibly published in 2020. The national data on the epidemic and testing used in the introduction is 3 years old. In the meantime the country did a population based survey in 2018 (KENPHIA) and the preliminary report is available now. I think the paper should use the updated data and cite current literature.

 The definition of resting is different for GF and KP. Though it is explained clearly in the method section, in other sections of the paper only one definition is used. This needs to be consistent.

 One of the limitations could be access to HIV testing services for populations in multiple sites. It could be possible that clients retested in other sites. How was that captured?

 The other limitation could be that KPs did not identify themselves as KPs in the testing sites due to stigma and discrimination and hence some of the KPs may have been categorised as GF in a public facility.

Is the work clearly and accurately presented and does it cite the current literature?

Partly

If applicable, is the statistical analysis and its interpretation appropriate?

I cannot comment. A qualified statistician is required.

Are all the source data underlying the results available to ensure full reproducibility?

Yes

Is the study design appropriate and is the work technically sound?

Yes

Are the conclusions drawn adequately supported by the results?

Yes

Are sufficient details of methods and analysis provided to allow replication by others?

Yes

Reviewer Expertise:

HIV prevention among key populations

I confirm that I have read this submission and believe that I have an appropriate level of expertise to confirm that it is of an acceptable scientific standard.

# Response

**Published:** 2021-01-26

**Authors:** Peter Mugo

**Affiliations:** Kenya Medical Research Institute, Kenya

***The paper is being reviewed and possibly published in 2020. The national data on the epidemic and testing used in the introduction is 3 years old. In the meantime the country did a population based survey in 2018 (KENPHIA) and the preliminary report is available now. I think the paper should use the updated data and cite current literature.***

 We thank the reviewer for pointing this out. The introduction has been updated with findings from the 2018 KENPHIA survey, also in response to a comment by reviewer 1. [Pages 5,6 (intro)]

***One of the limitations could be access to HIV testing services for populations in multiple sites. It could be possible that clients retested in other sites. How was that captured?***

 In the revised manuscript, we have clarified that clients were asked to report the date of their last HIV test, whether conducted at the same VCT or elsewhere. [Page 7 (data collection procedures)]

**The other limitation could be that KPs did not identify themselves as KPs in the testing sites due to stigma and discrimination and hence some of the KPs may have been categorised as GF in a public facility.**

 We have added this limitation, also in response to a comment by reviewer 1. [Page 23 (discussion; limitations)]

# Peer review: Reviewer response for version 1

**DOI:** 10.21956/wellcomeopenres.16834.r36589

**Published:** 2019-10-28

**Authors:** Mathieu Maheu-Giroux, Katia Giguère

**Affiliations:** 1 Department of Epidemiology, Biostatistics, and Occupational Health, School of Global and Population Health, McGill University, Montreal, Canada

In their research article, Dr. Peter M Mugo and colleagues used program data from HIV testing services to examine 2006-2007 trends in self-reported new diagnoses, first time testing, and time since last test in Kilifi county, Kenya. Importantly, the authors examined these trends among self-reported key population status of attendees.

 Overall, we found that the paper was innovative and well written. We congratulate the authors for leveraging the available wealth of testing data to gain a better understanding of longitudinal HIV testing trends. However, we have some concerns regarding limitations of these data and feel that some conclusions are not supported by the paper’s results.

 Please find our comments, suggestions, and questions below.

Methods 

- The most important limitation of the study is the use of self-reported information, especially with regards to new diagnoses. Several studies have found considerable under-reporting of knowledge of HIV status (see list at the end of review). We would recommend that the authors use extreme caution when interpreting these self-reported “new diagnoses”. Indeed, if one examines HIV national testing program data from several countries, it is difficult to reconcile the national total annual number of HIV positive tests performed with the estimated size of the population living with HIV without allowing for considerable re-testing of already diagnosed individuals. For example, in many countries the annual numbers of positive tests reported can represent up to 25 – 30% of the whole estimated PLHIV population, which is inconsistent with survey data on the proportion of PLHIV ever tested. For more details on these issues, the authors can consult this preprint (Maheu-Giroux et al. 2019 ^1^) (also in press at AIDS).
- A total of 47,893 tests encounters were recorded. Yet, the authors exclude almost half of these to obtain an “ *homogenous sample*”. It is unclear to us why this is warranted and how this achieves a sample that is more representative of the “ *at-risk adult population in the area*”. By definition, the authors’ data constitute a convenience sample (i.e., health-care seekers) that is probably not representative of the underlying “at-risk population”. Could the authors confirm that excluding 46% of observations does not change their main conclusion (qualitatively)?
- “ *HIV positivity*” is usually defined as the number of positive tests over the total number of HIV tests performed. Can the authors consider replacing what they call “ *HIV positivity*” with “ *% of self-reported new diagnosis*”? We understand this is a verbose change but it is also more accurate.
- It is said that risk behaviors were collected from 2010 onward. However, figures showing trends among key populations show estimates from 2009. Were risk behaviors rather collected from 2009?
- Why are the authors smoothing the time trends using LOWESS? Why is that warranted? Since their data do not constitute a sample – but rather contains all testing encounters in Kilifi county (akin to a census) – we are also wondering why the authors are using statistical test of significance to measure trends. If the proportion of self-reported first-time testers was 60% in the general population in 2006 and that it decreases to 20%ish in 2017 in Kilifi County, why perform statistical tests? The decrease was 40% point over that period: there are no random errors. Instead, it could be more appropriate for the authors to look at meaningful changes in testing metrics and disregard p-values to assess if changes are important.
- In the multivariable regression models, the inclusion of variables based on their statistical significance could be misleading. For example, a variable with a p-value>0.1 could become important after adjustments for other potential confounders. Could the authors include variables (and confounders, if any) that were determined *a priori* to plausibly be associated with the outcomes? The authors should, at the very least, recognized the limitations of their model-building process. (That being said, we appreciate the use of log-binomial models over logistic regressions.)
- Ethical approval was not sought because the data was de-identified. De-identification of individual-level observations that contains potentially sensitive information is usually not a sufficient reason for not obtaining ethics approval. Can the authors clarify? 

Results

- Figure 5: the authors conclude that there was no change over time for most of the outcomes presented in this figure. Actually, we see that some of the presented curves tend to be quadratic. It could be there was a (quadratic) change over time for some of these outcomes, but that the authors could not detect it because they tested a linear trend.
- It is written that, in 2017: “ *Overall FFT prevalence in KP was 28%” and “Overall late-retesting prevalence in KP was 83%”.* How could the sum of these two estimates be over 100%? Especially considering that we would expect that at least some of the tests in KP were on-time testing.  

Discussion 

- In the discussion section, it is written: “ *Annual retesting coverage in GP was 72% in 2017, which signals a population-level increase from the 2012 estimate of 55%. On the other hand, less than 20% of KP in the current study were retesting quarterly*.” Both estimates (72% and 20%) were not provided in the results section. Please make sure that discussed results are also presented in the results section.
- Some parts of the discussion are disconnected from the authors’ analyses. For example, the authors’ second paragraph of page 8 that discussed low educational attainment seems out of place. Why are the authors making statement such as “ *this suggests that HIV prevention programmes are insufficiently reaching those with low educational attainment*”? The authors only looked at HIV testing program data and HIV prevention programs are way broader than this. In fact, the whole paragraph is not supported by data presented in their article. The same applies for the discussion of the likely impact of “ *underage sex*”, “ *early childhood marriage*”, and “ *sex tourism*”. Could the authors avoid such wide extrapolations of their findings?
- The same applies for other sections of the discussion. For example, the authors conclude that PrEP users will likely need intense engagement and tailored support services in order to adhere to the recommended retesting frequency. This conclusion is surprising since the authors did not assess HIV testing among PrEP users. Please consider re-wording that section.
- It is written: “ *Testing coverage and HIV positivity varied by town […]”* Testing coverage was not assessed in this study. The only metric the authors are able to calculate are the % of first-time tester – but this is not testing coverage per se. You could well have a region where only 10% of the population has ever been tested for HIV but where repeat testing is very high among this 10% of “testers”. Even though the program data would show a very low proportion of first-time testers, testing coverage would still be <10%. Please refrain from making conclusions about testing coverage when examining program data only.
- A similar argument applies to the interpretation of the regression models of the factors associated with first-time testing. The fact that a certain variable (i.e., religion) is associated with self-reported first-time testing is not necessarily evidence that testing coverage is lower among this group.
- Among limitations of the study, please discuss also potential selection bias in assessment of trends due to the changes in covered populations in the different VCT centres over time. We also suggest discussing potential information bias from self-report “ *new diagnosis*” and, importantly, key population status. It is highly likely that a potential high proportion of MSM do not disclose same-sex activity to their health-care providers. The same applies for FSW.  

Minor comments 

- Introduction: The 2019 AIDS report published by UNAIDS has the updated figure for knowledge of HIV status in Kenya. Consider updating it.
- In the results section, it is written: “ *The percentage-point changes were similar for all sub-groups assessed*.” We think that it would worth mentioning “ *The percentage-point changes **per year** were similar*…”
- The conclusions regarding on-time testing would benefit from mentioning potential limitations associated with these self-reports. In particular, “ *telescoping bias*” is likely to occur if respondents inadvertently recall testing that occurred beyond the last 3 (KP) or 12 (GP) months. This would not necessarily affect the conclusion regarding trends but would affect the absolute proportion testing on time.
- Figure 4. There is a “T” missing at the beginning of the title.

Is the work clearly and accurately presented and does it cite the current literature?

Partly

If applicable, is the statistical analysis and its interpretation appropriate?

No

Are all the source data underlying the results available to ensure full reproducibility?

Yes

Is the study design appropriate and is the work technically sound?

Yes

Are the conclusions drawn adequately supported by the results?

No

Are sufficient details of methods and analysis provided to allow replication by others?

Partly

Reviewer Expertise:

Infectious disease epidemiology; HIV and other sexually transmitted and blood-borne infections; Impact Evaluation; Mathematical modeling; Population health.

We confirm that we have read this submission and believe that we have an appropriate level of expertise to state that we do not consider it to be of an acceptable scientific standard, for reasons outlined above.

## References

[1] : National HIV testing and diagnosis coverage in sub-Saharan Africa: a new modeling tool for estimating the “first 90” from program and survey data. *bioRxiv* .2019; 10.1101/532010 10.1101/532010 PMC691923531764066

[2] : Performance of self-reported HIV status in determining true HIV status among older adults in rural South Africa: a validation study. *Journal of the International AIDS Society* .2017;20(1) : 10.7448/IAS.20.1.21691 10.7448/IAS.20.1.21691 PMC557773428782333

[3] : Monitoring progress towards the first UNAIDS target: understanding the impact of people living with HIV who re-test during HIV-testing campaigns in rural Mozambique. *J Int AIDS Soc* .21(4) : 10.1002/jia2.25095 e25095 10.1002/jia2.25095 29652098PMC5898226

[4] : Reliability, sensitivity and specificity of self-report of HIV test results. *AIDS Care* .2007;19(5) : 10.1080/09540120601087004 692-6 10.1080/09540120601087004 17505932

[5] : Nondisclosure of HIV status in a clinical trial setting: antiretroviral drug screening can help distinguish between newly diagnosed and previously diagnosed HIV infection. *Clin Infect Dis* .2014;58(1) : 10.1093/cid/cit672 117-20 10.1093/cid/cit672 24092804PMC3864502

[6] : Beyond Social Desirability Bias: Investigating Inconsistencies in Self-Reported HIV Testing and Treatment Behaviors Among HIV-Positive Adults in North West Province, South Africa. *AIDS Behav* .2018;22(7) : 10.1007/s10461-018-2155-9 2368-2379 10.1007/s10461-018-2155-9 29779162PMC13310556

# Response

**Published:** 2021-01-26

**Authors:** Peter Mugo

**Affiliations:** Kenya Medical Research Institute, Kenya

***The most important limitation of the study is the use of self-reported information, especially with regards to new diagnoses. Several studies have found considerable under-reporting of knowledge of HIV status (see list at the end of review). We would recommend that the authors use extreme caution when interpreting these self-reported “new diagnoses”. Indeed, if one examines HIV national testing program data from several countries, it is difficult to reconcile the national total annual number of HIV positive tests performed with the estimated size of the population living with HIV without allowing for considerable re-testing of already diagnosed individuals. For example, in many countries the annual numbers of positive tests reported can represent up to 25 – 30% of the whole estimated PLHIV population, which is inconsistent with survey data on the proportion of PLHIV ever tested. For more details on these issues, the authors can consult this preprint (Maheu-Giroux et al. 2019) (also in press at AIDS).***

 We agree with the reviewer that not all positive tests at the VCT may be new diagnoses. We excluded from our analysis clients seeking confirmatory testing, after testing positive elsewhere.  We have revised the title and the manuscript to use the term “HIV positivity” and speak of positive test results and not new diagnosis. We have also added this limitation and referenced the study that the reviewer kindly pointed us to in the discussion. [Page 1 (title), 5 (abstract), 6 (intro), 18 (factors associated with HIV positivity), 21-23 (discussion)]

***A total of 47,893 tests encounters were recorded. Yet, the authors exclude almost half of these to obtain an “homogenous sample”. It is unclear to us why this is warranted and how this achieves a sample that is more representative of the “at-risk adult population in the area”. By definition, the authors’ data constitute a convenience sample (i.e., health-care seekers) that is probably not representative of the underlying “at-risk population”. Could the authors confirm that excluding 46% of observations does not change their main conclusion (qualitatively)?***

 Our sample selection was guided by our goal to assess trends in “adult walk-in VCT clients”, i.e., clients seeking testing out of their own initiative and not those testing as a result of outreach activities. Clients mobilized during outreach activities may be more open to social pressure to test or retest, and differences in the frequency and intensity of outreach activities over the years would make time trends difficult to assess.

 In response to this comment, we have added supplementary table 1 with data on the numbers of encounters and HIV positivity of the categories excluded. [Pages 7, 8 (Data collection procedures and sample selection)]

***“HIV positivity” is usually defined as the number of positive tests over the total number of HIV tests performed. Can the authors consider replacing what they call “HIV positivity” with “% of self-reported new diagnosis”? We understand this is a verbose change but it is also more accurate.***

 Our calculation of positivity was as per the reviewer’s definition, i.e., “number of positive tests divided by total number of HIV tests performed”. We have changed the definition to reflect this, viz: “proportion of test encounters where the result was positive”. In the revised manuscript, we have avoided referring to “new diagnosis,” in response to this reviewer’s earlier comment. [Pages 1 (title), 5 (abstract), 6 (intro), 8 (data analysis), 18 (factors associated with HIV positivity), 21-23 (discussion).}

***Why are the authors smoothing the time trends using LOWESS? Why is that warranted?***

 We smoothed the plots to improve visibility of the trends, especially for graphs where we have plots that cross each other. We have added in the supplementary materials the actual data points on which the plots are based. [Pages 11 (figure 2),12 (figure 3 and 4) and 13 (figure 5). Supplementary tables 2- 9.]

***Since their data do not constitute a sample – but rather contains all testing encounters in Kilifi county (akin to a census) – we are also wondering why the authors are using statistical test of significance to measure trends. If the proportion of self-reported first-time testers was 60% in the general population in 2006 and that it decreases to 20%ish in 2017 in Kilifi County, why perform statistical tests? The decrease was 40% point over that period: there are no random errors. Instead, it could be more appropriate for the authors to look at meaningful changes in testing metrics and disregard p-values to assess if changes are important.***

 In response to this comment, we have omitted the test for trend. As our goal was to describe the change over the years and not simply the start and end points, we now focus, as suggested by the reviewer, on the slope (percentage-point change per year). [Pages 5 (abstract), 8 (data analysis), 11, 12, 13 (time trends in FTT, late retesting and positivity), 21, 22 (discussion)]

***In the multivariable regression models, the inclusion of variables based on their statistical significance could be misleading. For example, a variable with a p-value>0.1 could become important after adjustments for other potential confounders. Could the authors include variables (and confounders, if any) that were determined a priori to plausibly be associated with the outcomes? The authors should, at the very least, recognized the limitations of their model-building process. (That being said, we appreciate the use of log-binomial models over logistic regressions.)***

 We thank the reviewer for commending our use of log-binomial regression which gives estimates that are more intuitive.

 We did not initially highlight our a priori variables since these variables met criteria of p<0.1 in bivariable analysis and so were included in the full multivariate model. However, sex and age were considered confounders a priori; this is now clarified in the manuscript.

 The issue of variables being excluded because of p>0.1 in bivariable analysis that could have had P<0.1 in adjusted analysis does not arise for our study since, as explained here above, all variables were included in the multivariable model. [Pages 8, (data analysis)]

***Ethical approval was not sought because the data was de-identified. De-identification of individual-level observations that contains potentially sensitive information is usually not a sufficient reason for not obtaining ethics approval. Can the authors clarify?***

 Ethical approval for the analysis has since been granted by the KEMRI Scientific and Ethical Review Unit (SERU). We have amended the ethical statement to reflect the same. [Page 8 (ethical statement)]

***Figure 5: the authors conclude that there was no change over time for most of the outcomes presented in this figure. Actually, we see that some of the presented curves tend to be quadratic. It could be there was a (quadratic) change over time for some of these outcomes, but that the authors could not detect it because they tested a linear trend.***

 We have omitted the statistical test for trend and now focus on the estimated slope (percentage-point change per year), also in response to another comment above. [Pages 11 (figure 2),12 (figure 3 and 4) and 13 (figure 5).]

**It is written that, in 2017: “ *Overall FFT prevalence in KP was 28%” and “Overall late-retesting prevalence in KP was 83%”.* How could the sum of these two estimates be over 100%? Especially considering that we would expect that at least some of the tests in KP were on-time testing.**

 FTT is based on data from clients who reported never testing before, while late retesting is from clients who ever tested before, so the numerators and denominators for these two metrics are different. We have edited the text for clarity, as these statistics refer to the proportion of all encounters that involved first-time testing or late-retesting *within subgroups*. [Page 8 (data analysis)]

***In the discussion section, it is written: “Annual retesting coverage in GP was 72% in 2017, which signals a population-level increase from the 2012 estimate of 55%. On the other hand, less than 20% of KP in the current study were retesting quarterly.” Both estimates (72% and 20%) were not provided in the results section. Please make sure that discussed results are also presented in the results section.***

 We thank the reviewer for pointing out this problem. We edited the discussion to ensure that it only includes results that were presented in the results section. [Page 21 (discussion)]

***Some parts of the discussion are disconnected from the authors’ analyses. For example, the authors’ second paragraph of page 8 that discussed low educational attainment seems out of place. Why are the authors making statement such as “this suggests that HIV prevention programmes are insufficiently reaching those with low educational attainment”? The authors only looked at HIV testing program data and HIV prevention programs are way broader than this. In fact, the whole paragraph is not supported by data presented in their article. The same applies for the discussion of the likely impact of “underage sex”, “early childhood marriage”, and “sex tourism”. Could the authors avoid such wide extrapolations of their findings?***

 We thank the reviewer for the comment and critical review of our discussion points. The discussion section has been amended to reflect potential strategies based on literature to tackle the socioeconomic inequalities such as poverty and education and their impact on HIV. [Pages 21-23 (discussion)]

***It is written: “Testing coverage and HIV positivity varied by town […]” Testing coverage was not assessed in this study. The only metric the authors are able to calculate are the % of first-time tester – but this is not testing coverage per se. You could well have a region where only 10% of the population has ever been tested for HIV but where repeat testing is very high among this 10% of “testers”. Even though the program data would show a very low proportion of first-time testers, testing coverage would still be <10%. Please refrain from making conclusions about testing coverage when examining program data only.***

 We thank the reviewer for pointing this out. In the revised manuscript, we have avoided the use of “coverage” and “prevalence” when referring to the testing encounters were studied. [Page 23 (discussion)]

***Among limitations of the study, please discuss also potential selection bias in assessment of trends due to the changes in covered populations in the different VCT centres over time. We also suggest discussing potential information bias from self-report “new diagnosis” and, importantly, key population status. It is highly likely that a potential high proportion of MSM do not disclose same-sex activity to their health-care providers. The same applies for FSW.***

 We thank the reviewer for the comment and critical review of limitations. We edited the limitations to discuss potential social desirability bias that may lead some clients to report their previous test as negative even if it was positive. In addition, we have commented on the effects of stigma and discrimination towards MSM which may result in under-reporting of same-sex behavior practices among men, and under-reporting of transactional sex among women. We have also discussed the limitation of cross-site comparisons of time trends that may have been biased by changes in covered populations in the different VCTs over time. [Page 25 para 2]
